# Lipases of germinating jojoba seeds efficiently hydrolyze triacylglycerols and wax esters and display wax ester-synthesizing activity

**DOI:** 10.1186/s12870-020-02823-4

**Published:** 2021-01-19

**Authors:** Adam Kawiński, Magdalena Miklaszewska, Szymon Stelter, Bartosz Głąb, Antoni Banaś

**Affiliations:** 1 Intercollegiate Faculty of Biotechnology, University of Gdansk and Medical University of Gdansk, Abrahama 58, 80–307, Gdańsk, Poland; 2 Department of Plant Physiology and Biotechnology, Faculty of Biology, University of Gdańsk, Wita Stwosza 59, 80-308 Gdańsk, Poland

**Keywords:** Wax esters, Lipase, Wax ester hydrolase, *Simmondsia chinensis*, Jojoba, Wax ester synthesis, Triacylglycerols

## Abstract

**Background:**

*Simmondsia chinensis* (jojoba) is the only plant known to store wax esters instead of triacylglycerols in its seeds. Wax esters are composed of very-long-chain monounsaturated fatty acids and fatty alcohols and constitute up to 60% of the jojoba seed weight. During jojoba germination, the first step of wax ester mobilization is catalyzed by lipases. To date, none of the jojoba lipase-encoding genes have been cloned and characterized. In this study, we monitored mobilization of storage reserves during germination of jojoba seeds and performed detailed characterization of the jojoba lipases using microsomal fractions isolated from germinating seeds.

**Results:**

During 26 days of germination, we observed a 60–70% decrease in wax ester content in the seeds, which was accompanied by the reduction of oleosin amounts and increase in glucose content. The activity of jojoba lipases in the seed microsomal fractions increased in the first 50 days of germination. The enzymes showed higher activity towards triacylglycerols than towards wax esters. The maximum lipase activity was observed at 60 °C and pH around 7 for triacylglycerols and 6.5–8 for wax esters. The enzyme efficiently hydrolyzed various wax esters containing saturated and unsaturated acyl and alcohol moieties. We also demonstrated that jojoba lipases possess wax ester-synthesizing activity when free fatty alcohols and different acyl donors, including triacylglycerols and free fatty acids, are used as substrates. For esterification reactions, the enzyme utilized both saturated and unsaturated fatty alcohols, with the preference towards long chain and very long chain compounds.

**Conclusions:**

In in vitro assays, jojoba lipases catalyzed hydrolysis of triacylglycerols and different wax esters in a broad range of temperatures. In addition, the enzymes had the ability to synthesize wax esters in the backward reaction. Our data suggest that jojoba lipases may be more similar to other plant lipases than previously assumed.

**Supplementary Information:**

The online version contains supplementary material available at 10.1186/s12870-020-02823-4.

## Background

Jojoba (*Simmondsia chinensis* Link, Buxaceae) is a perennial shrub that grows naturally in the deserts of southwestern North America. It is the only known plant species which accumulates wax esters in the seeds as storage reserves instead of triacylglycerols. Wax esters constitute up to 60% of seed weight and are composed of very-long-chain monounsaturated fatty acids and alcohols, such as eicosenoic acid (20:1), docosenoic acid (22:1), eicosenol (20:1-OH) and docosenol (22:1-OH) [1, 2]. Apart from lipids, jojoba seeds are composed of proteins (15% of fresh weight), carbohydrates and starch (10%), water (5–10%), and seed coat (5–10%) [3, 4]. Wax esters are stored in lipid droplets (frequently called wax bodies), spherical storage organelles with a diameter of 1 to 1.5 μm, which localize in cotyledons [5–8]. Major surface proteins of wax bodies include oleosins, caleosins, steroleosins and lipid droplet-associated proteins (LDAPs) [8]. Jojoba wax esters are valuable compounds with numerous important commercial applications in the cosmetic and pharmaceutical industry, and are difficult to synthesize chemically. *S. chinensis* is currently cultivated for its oil in many countries, including USA, Mexico, Chile, Argentina, India, Australia, and Egypt [9]. Since the jojoba cultivation is both relatively challenging and low yielding, in recent years jojoba-like wax esters synthesis was established in bacteria [10], yeasts [11, 12], and oilseed plants, such as *Camelina sativa* and *Crambe abyssinica* [13–16]. However, the transgenic plants with high amounts of wax esters in their seeds had a decreased frequency of seed germination and disturbed early seedling growth [14, 16, 17]. It was suggested that impaired wax ester degradation may be a bottleneck for seed viability in high-wax esters accumulating plants [18].

In oilseed plants, the mobilization of storage lipids is well understood. Triacylglycerols (TAGs) accumulated in seed lipid droplets are hydrolyzed during germination by TAG lipases (EC 3.1.1.3), which cleave the ester bond between a fatty acid and a glycerol backbone [19]. The glycerol is converted to dihydroxyacetone phosphate (DHAP) and enters gluconeogenesis. Free fatty acids are transported to peroxisomes where they are broken down to acetyl-CoA via β-oxidation. Acetyl-CoA is further metabolized by the glyoxylate cycle to four-carbon organic acids, which can be used to produce sugars in gluconeogenesis [20, 21]. TAG lipases have been identified in a large number of plant species, including oilseed crops such as *Brassica napus* or *Jatropha curcas*. The enzymes characterized so far showed different properties, depending on the plant species. For example, the optimal pH ranged from 4 to 11 and temperature from 25 °C to 80 °C. In addition, lipases are capable of catalyzing both hydrolysis and synthesis reactions [22]. Due to their biochemical properties, wide availability from natural sources and low production costs, plant lipases are considered to be potential candidates for industrial applications, especially in food, pharmaceutical, and biofuel industries [22, 23].

In contrast to TAG hydrolysis, wax ester mobilization in germinating jojoba seeds requires activity of three unique enzymes, associated with membranes of wax bodies. First, wax esters are hydrolyzed by a jojoba lipase (wax ester hydrolase, WEH, EC 3.1.1.50) to fatty acids and fatty alcohols. Then, fatty alcohols are converted to fatty acids through oxidation pathway comprising two enzymes: a fatty alcohol oxidase (FAO) and a fatty aldehyde dehydrogenase (FADH) [24]. The nascent fatty acids enter β-oxidation and are further metabolized to sucrose by the metabolic pathways similar to triacylglycerol-storing plant species [4, 6].

To date, only jojoba FAO and FADH were isolated and characterized. Biochemical properties of both enzymes, including activity towards very long chain fatty alcohols and fatty aldehydes, and high expression of their genes in germinating jojoba seeds confirmed that FAO and FADH are involved in fatty alcohol oxidation pathway in jojoba [24]. The knowledge on possible molecular identity of jojoba lipases is still scarce. In the recently published jojoba genome, more than 100 genes of the putative lipases were reported [8]. Further studies are needed to elucidate the functions of newly identified enzymes.

The available data on the biochemical properties of jojoba lipases were obtained in the late 1970s by Huang and Moreau. The activity of the enzyme was detected in 20-day-old jojoba seedlings and was associated with membranes of wax bodies [4]. It was shown that wax ester hydrolase activity increased drastically during the first 15 days of germination. The enzyme had optimal activity at pH from 8.5 to 9.0 and efficiently hydrolyzed monoacylglycerols and wax esters, while the activity towards diacylglycerols and triacylglycerols was low [25].

The aim of this study was to get a deeper insight into the characteristics of the jojoba lipases activity in germinating jojoba seeds and to perform a more detailed analysis of their substrate specificity. Here, we also report wax ester-synthesizing activity of jojoba lipases.

## Results

### Mobilization of jojoba seed reserves during germination

**Fig. 1:**
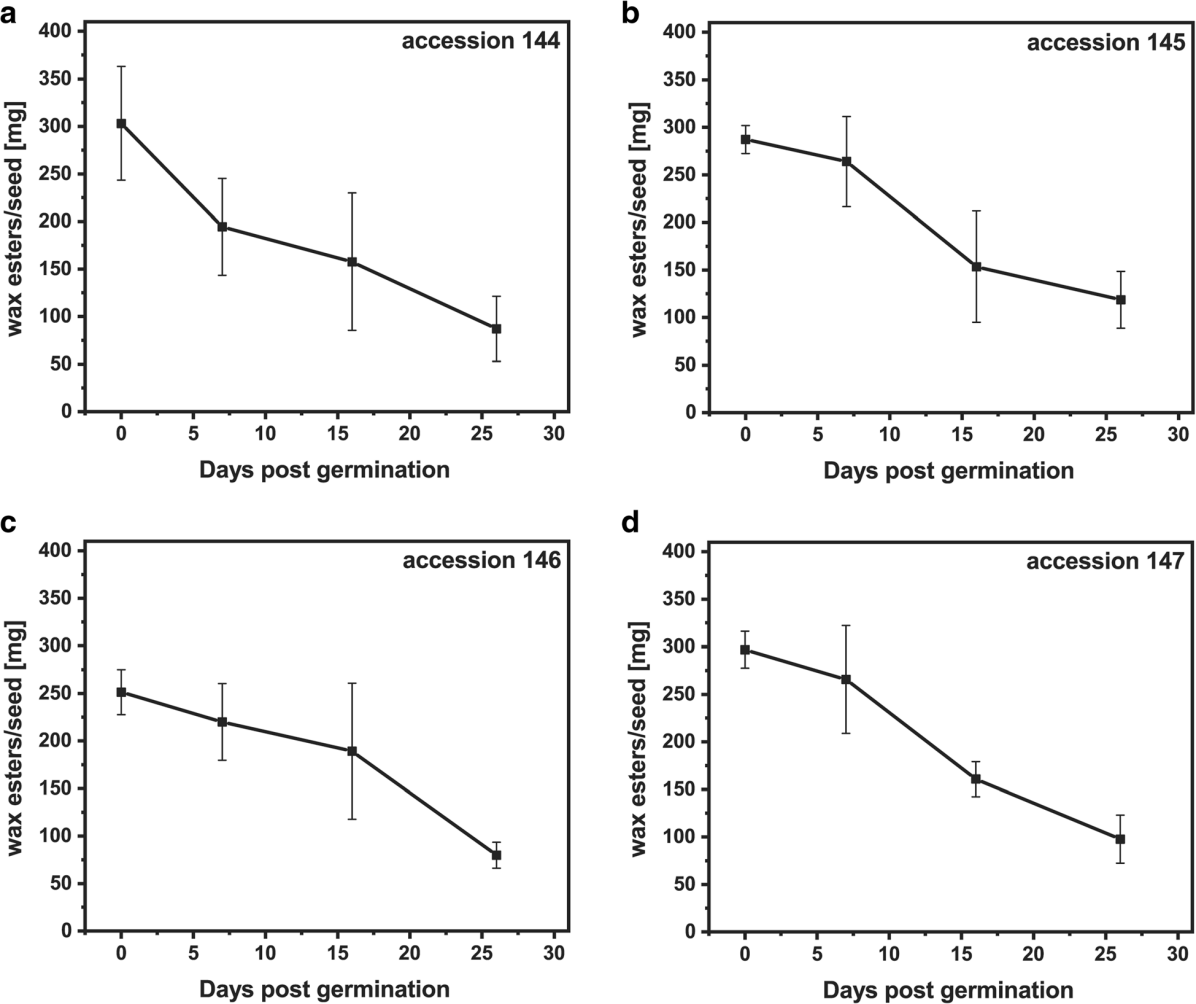
Wax ester mobilization during jojoba seed germination. Changes in wax ester content in germinating jojoba seeds of accession 144 (**a**), accession 145 (**b**), accession 146 (**c**), accession 147 (**d**). Data represent the mean of four biological replicates and error bars show standard deviation

**Fig. 2:**
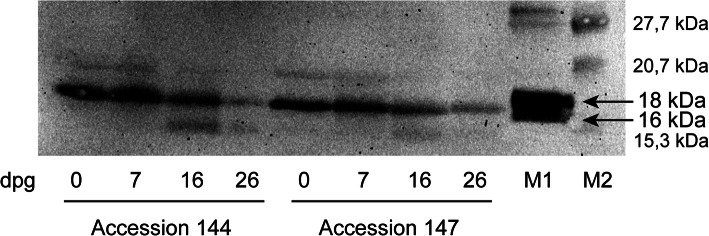
Immunoblot analysis of oleosin content in total protein extracts of jojoba seeds during germination. M1: oleosins from maize, M2: protein marker. Numbers on the right indicate the positions of molecular mass markers. The arrows indicate two isoforms of the maize oleosins. Similar changes were observed for accessions 145 and 146. The full-length blot is presented in Additional file 1: Fig. S6

**Fig. 3:**
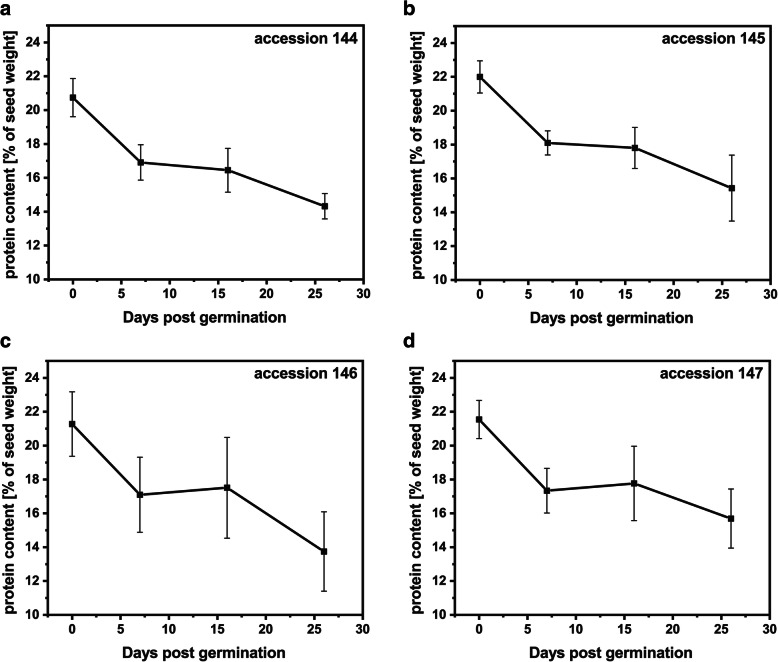
Protein mobilization during jojoba seed germination. Changes in protein content (relative to the seed initial weight) in germinating jojoba seeds of accession 144 (**a**), accession 145 (**b**), accession 146 (**c**), accession 147 (**d**). Data represent the mean of four biological replicates and error bars show standard deviation

Jojoba seeds from four different accessions were germinated for a period of 26 days (Additional file 1: Fig. S1), and changes in wax ester content were monitored using GC-FID analysis. In four examined accessions, the wax ester content was reduced by 60–70% in the first 26 days of germination (Fig. 1). The major decrease was observed in the first week of germination for accession 144 (36%; Fig. 1a), and between 7 and 16 days post germination (dpg) for accessions 145 and 147 (approximately 40%; Fig. 1b, d). For accession 146, the highest drop in wax ester content (by 58%) occurred between 16 and 26 dpg (Fig. 1c). The mobilization of wax esters was accompanied by the increase in glucose and starch content during germination (Additional file 1: Fig. S2) and the decrease in oleosin amounts in protein extracts from germinating jojoba seeds, as shown in the representative immunoblot for accessions 144 and 147 in Fig. 2. The initial protein content was approximately 21% of seed weight, and in the first 26 days of germination decreased to approximately 15% for all accessions. The degradation occurred mostly within the first week of germination (Fig. 3).

**Fig. 4:**
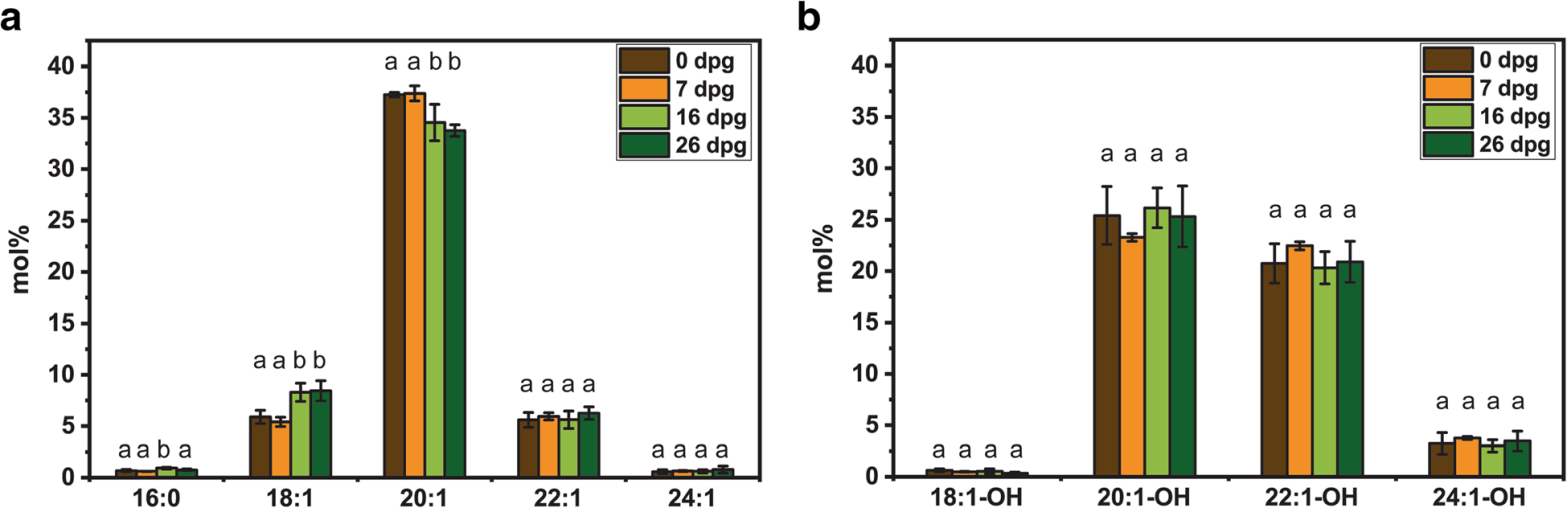
Fatty acid and fatty alcohol composition of germinating jojoba seeds. The relative content of main fatty acids (FA; **a**) and fatty alcohols (FA-OH; **b**) in lipids of jojoba seeds during germination (% of total FA and FA-OH). Data represent the mean of four biological replicates (accession 145) and error bars show standard deviation. The different letters denote statistical significance between FA or FA-OH content at different stages of germination (one-way ANOVA followed by Tukey’s post-hoc test, *p* < 0.05, *n* = 4)

Analysis of lipid classes in jojoba seeds from four accessions revealed that wax esters are predominant class of lipids (95–98%). Polar lipids, TAGs, free fatty acids and free alcohols comprise approximately 1–2%, 0.4–1%, 0.3–1% and 0.2–1%, respectively. In the mature seeds of all tested accessions, the dominating fatty acids were: 20:1 (about 36% of total amount of fatty acids and fatty alcohols), 18:1 (about 7%), 22:1 (about 6%), 16:0 (about 0.8%), 24:1 (about 0.5%), whereas the most abundant fatty alcohols were: 20:1-OH (about 25% of total amount of fatty acids and fatty alcohols), 22:1-OH (about 21%), 24:1-OH (about 3%), 18:1-OH (0.7%) (Additional file 2: Table S1). The pattern of changes in fatty acid and fatty alcohol relative content during 26 days of germination was similar for all accessions. A representative set of results for accession 145 is presented in Fig. 4. A significant decrease of 20:1 and an increase of 18:1 content was observed during germination in all accessions.

### Lipases activity in microsomal fractions of germinating jojoba seeds

Since the genes encoding jojoba lipases have not yet been successfully cloned, we used microsomal membrane fractions isolated from jojoba seeds at different stages of germination to test the activity of membrane associated lipases. In our preliminary analyses, we tested lipase activity in microsomes isolated at 0, 14, 35 and 50 dpg using [^14^C] wax esters ([^14^C]WEs) and [^14^C] triacylglycerols ([^14^C]TAGs) as substrates. At very early stages of germination, the lipase activity towards both [^14^C]18:1-TAG and [^14^C]20:1–18:1 WE was very low, and increased significantly during germination (Additional file 2: Table S2). We also observed an increase in oleosin content in the microsomal membrane fractions during 7–26 dpg (Additional file 1: Fig. S3). For further studies, the microsomal fraction with high lipase activity was used.

### Biochemical characterization of jojoba lipases

**Fig. 5:**
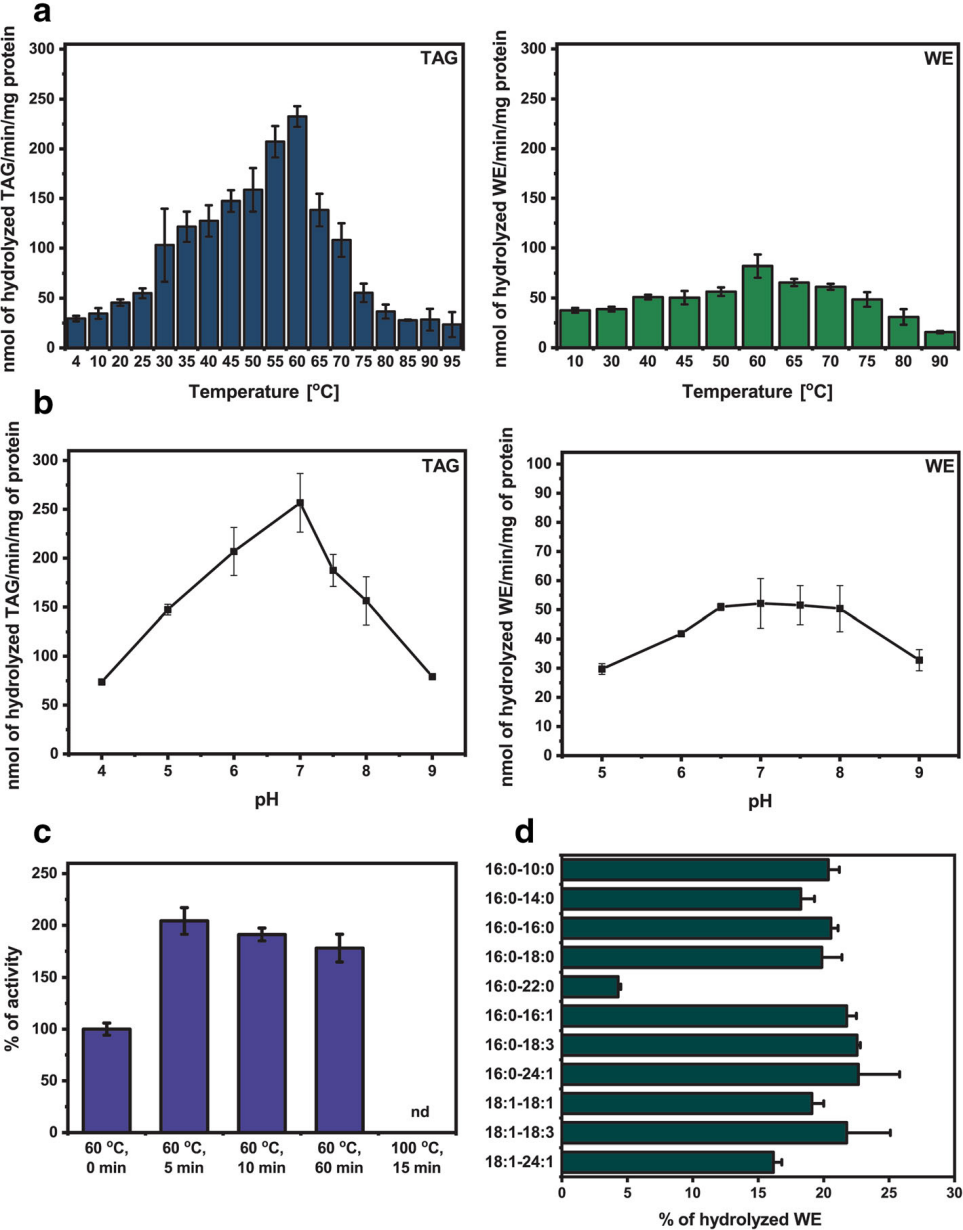
Biochemical characterization of the jojoba seed lipase activity . **a**. The impact of temperature on the jojoba seed lipase activity towards 18:1-TAG (left panel) and 18:1–18:1 WE (right panel). Data represent mean values and error bars show the range of duplicates. Assay conditions: aliquots (2.5 nmol of endogenous PC) of microsomal fraction mixture isolated from two individual jojoba seeds from each accession (35 dpg); 20 nmol of [^14^C]18:1-TAG or [^14^C]18:1–18:1 WE added to freeze-dried microsomes in 19 μl benzene; benzene evaporation, and addition of 100 μl 0.1 HEPES buffer (pH 7.0); incubation for 2.5 min (for TAG) or 1.5 min (for WE). **b**. The impact of the pH on the jojoba seed lipase activity towards 18:1-TAG (left panel) and 18:1–18:1 WE (right panel). Data represent mean values and error bars show the range of duplicates. Assay condition: as above with 0.1 M citrate buffer (pH 4.0–5.0), phosphate buffer (pH 6.0–8.0), or Tris-HCl buffer (9.0), 5 min incubation at 35 °C. **c**. The impact of pre-incubation conditions on jojoba seed lipase activity towards 18:1-TAG. Data represent mean values and error bars show the range of duplicates; nd – not detected. Assay conditions: as above, 5 min incubation at 60 °C. **d**. Substrate specificity of jojoba seed lipases towards saturated and unsaturated WEs. Data represent mean values and error bars show the range of duplicates. Assay condition: as above with 2.5 nmol of [^14^C] WE, 10 min incubation at 60 °C.

Since the jojoba lipases showed activity towards TAGs and WEs, we used both substrates for biochemical characterization of the enzymes. The activity towards 18:1-TAG was approximately three-fold higher than towards 18:1–18:1 WE (Fig. 5a). The jojoba lipases present in the microsomal fractions proved to be resistant to physical and chemical conditions. They remained active in a broad range of temperatures and pH. A gradual increase in lipase activity towards 18:1-TAG was observed with an increase of temperature from 4 °C to 60 °C. Further increase to 95 °C resulted in 70% reduction of the enzyme activity (Fig. 5a). In the case of 18:1–18:1 WE, the lipase activity at 10 °C was already nearly at 50% of its maximum level, which was observed at 60 °C (Fig. 5a). In addition, the preincubation of the microsomes at 60 °C up to 1 h before performing the enzymatic assays enhanced the lipase activity (Fig. 5c).

The optimal lipase activity towards 18:1-TAG was observed at pH ranging from 6 to 7 and decreased two to three fold when pH was lower than 6 or higher than 7 (Fig. 5b). For WEs, the highest activity was observed at pH between 6.5 and 8 (Fig. 5b). Based on obtained results, we used pH 7.0 and 60 °C for further studies as in these conditions lipases were the most active. Furthermore, two different reaction buffers were tested. Since the activity of lipases was higher in HEPES buffer, it was used to study substrate specificity (Additional file 1: Fig. S4). The addition of cations, such as Mg^2+^ and Ca^2+^, stimulated the lipase activity (with approximately a 2-fold increase) (Additional file 1: Fig. S5).

To study the substrate specificity of jojoba lipases, we used different wax esters containing saturated and unsaturated acyl and alcohol moieties. We observed no significant differences in the substrate specificity for wax esters containing saturated (16:0-FA) or monounsaturated (18:1-FA) fatty acids. Except for the long chain saturated wax esters (16:0–22:0), all other wax ester compounds were efficiently hydrolyzed, without significant differences (Fig. 5d).

### Wax ester-synthesizing activity of jojoba lipases

**Fig. 6:**
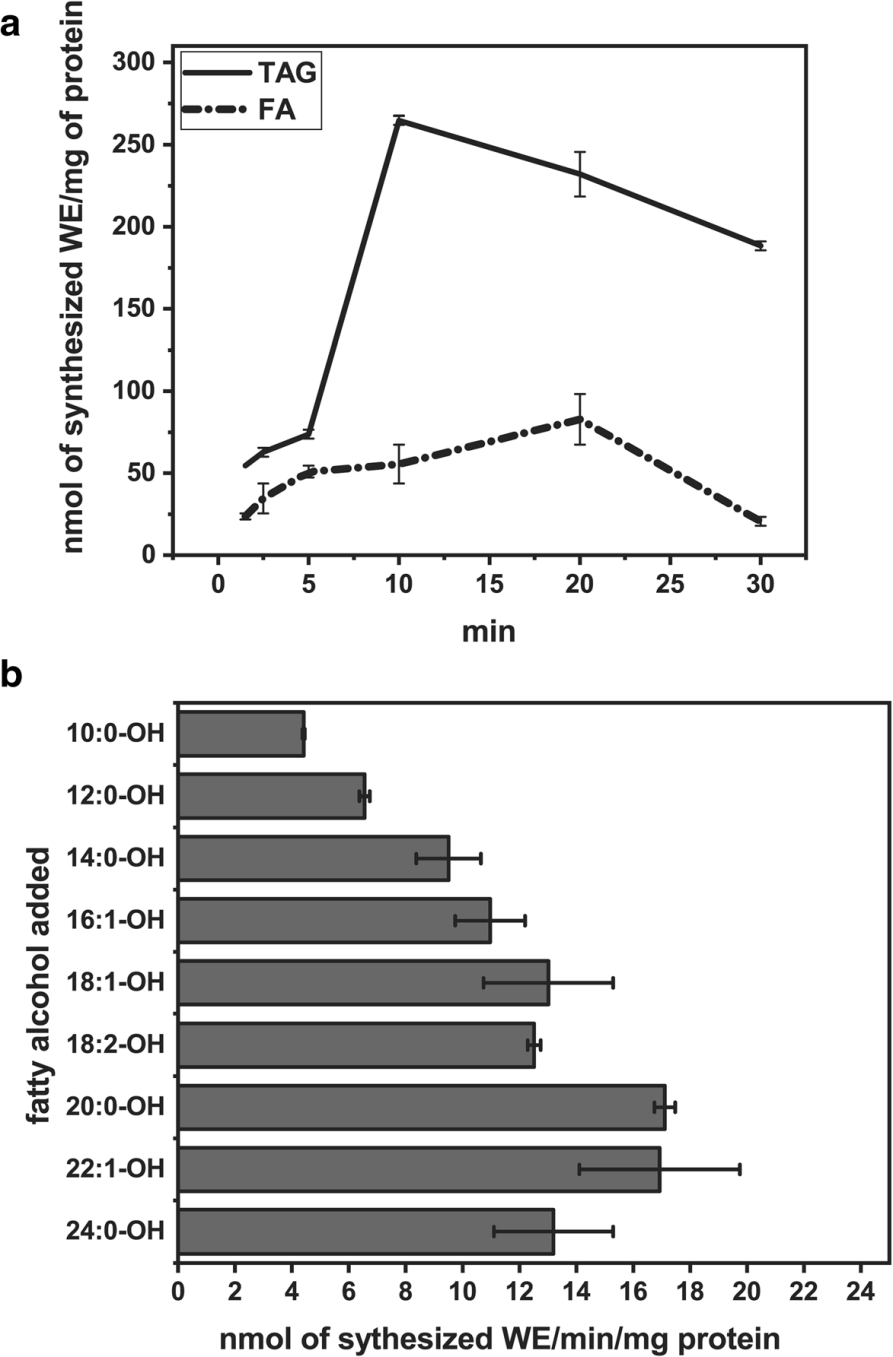
WE-synthesizing activity of the jojoba seed lipases. **a**. Time course of WE synthesis from 18:1-TAG and 18:1-FA combined with 18:1-OH. Data represent mean values and error bars show the range of duplicates. Assay condition: aliquots (2.5 nmol of endogenous PC) of microsomal fraction mixture (isolated from two individual jojoba seeds from each accession, 35 dpg); substrates (20 nmol of [^14^C]18:1-FA or [^14^C]18:1-TAG and 20 nmol of fatty alcohol) added to freeze-dried microsomes in 19 μl benzene; benzene evaporation, and addition of 100 μl 0.1 M HEPES buffer (pH 7.0); incubation at 60 °C. **b**. WE synthesis from 18:1-FA and different fatty alcohols. Data represent mean values and error bars show the range of duplicates. Assays conditions: as above, 20 min incubation at 60 °C

Apart from the ability to hydrolyze both WEs and TAGs, the lipases present in the utilized microsomal fractions revealed the wax ester-synthesizing activity from different acyl donors and free fatty alcohols delivered to the microsomal fractions. WEs were efficiently synthesized from TAGs, diacylglycerols (DAGs), and free fatty acids, while no WE synthesis from fatty acyl-CoAs and phospholipids could be detected (Table 1, Fig. 6a). WE synthesis was also observed when only free fatty alcohol was added to the reaction mixture, indicating that the enzyme used endogenous acyl-donors (Table 1). When neutral lipids were removed from microsomal fractions using acetone, addition of TAG was necessary to obtain WEs synthesis. The amount of wax esters synthesized from 18:1-TAG in combination with 18:1-OH was approximately 3-fold higher compared with a combination of 18:1-FA with 18:1-OH (Fig. 6a). The synthesis of WEs was the most efficient for long fatty alcohols with no discrimination against saturated or unsaturated fatty alcohols in combination with [^14^C]18:1-FA. The highest activity was observed for long chain and very long chain fatty alcohols (Fig. 6b). When lipase inhibitor (tetrahydrolipstatin) was present, we observed no wax ester synthesis from added [^14^C]18:1-FA and fatty alcohols. Tetrahydrolipstatin also efficiently inhibited the hydrolysis of wax esters and TAG in lipase activity assays. When DTNB (which combines CoAs and ACPs) was added to the assay, the amount of wax esters synthesized de novo was not lower than in the control, which shows that added [^14^C]18:1-FA was not activated to acyl-CoA or acyl-ACP before utilization for WE synthesis (Table 2).

**Table 1:** Wax ester synthesis by the microsomal fractions of germinated jojoba seeds from different substrates

	Substrates added (1 nmol of each one/assay)									
	[^14^C]18:1-OH			Tri-[^14^C]18:1-TAG			18:1-OH			
	–	+ 18:1-DAG	+ 18:1-TAG	–	+ 18:1-OH	+ cholesterol	+ [^14^C]18:1-FA	+ [^14^C]18:1-CoA	+ [^14^C]PC	+ [^14^C]PE
[^14^C]WE formed [pmol]	167 ± 19	284* ± 27	248* ± 22	76 ± 13	163* ± 7	61 ± 6	65 ± 9	0.0	0.0	0.0
[^14^C]WE formed [% of control]	100	159	139	100	214	80	-	-	-	-

^*^ – significant difference between control assays with only [^14^C] substrate added and assays with two substrates (*t* test, *p <* 0.01, *n* = 4)

Data represent the mean of four biological replicates ± standard deviation. Assay conditions: aliquots (2.5 nmol of endogenous PC) of microsomal fractions of germinated jojoba seed (35 dpg); substrates added to freeze dried microsomes in 19 μl benzene; benzene evaporation and addition of 100 μl 0.1 M phosphate buffer (pH 7.2); 15 min incubation at 35 °C

**Table 2:** Effect of tetrahydrolipstatin and DTNB on wax ester synthesis by the microsomal fractions of germinated jojoba seeds from different substrates

Substrates	WE synthesis (nmol/min/mg protein)		
	control	tetrahydrolipstatin	DNTB
[^14^C]18:1-FA + 12:0-OH	8.5 ± 1.1	0	9.2 ± 2.5
[^14^C]18:1-FA + 18:1-OH	16.7 ± 1.5	0	18.8 ± 4.1
[^14^C]18:1-FA + 20:0-OH	13.3 ± 0.4	0	19.3 ± 2.2

Data represent mean values and the range of duplicates. Assay conditions: aliquots (2.5 nmol of endogenous PC) of microsomal fraction mixture (isolated from two individual jojoba seeds from each accession, 35 dpg); substrates (20 nmol of each) added to freeze dried microsomes in 19 μl benzene; benzene evaporation and addition of 100 μl 0.1 M HEPES buffer (pH 7.0) containing 20 μM tetrahydrolipstatin or 7 mM DTNB; 10 min incubation at 60 °C

## Discussion

Jojoba is a unique plant that accumulates wax esters as storage lipids, which during germination are metabolized to carbohydrates [6]. We observed 60–70% drop in wax ester content during the first 26 days of germination in all four tested jojoba accessions, which is in line with the previous reports [4, 26]. The profile of wax ester hydrolysis varied among different jojoba accessions, but generally the highest decrease in wax ester content occurred after 7 dpg. The reduction of eicosenoic acid (20:1) relative content during germination suggests that wax esters containing this fatty acid may be mobilized at higher rate compared with wax esters containing 18:1 or 22:1 as acyl moieties. Wax ester species containing 20:1 are the predominant ones in jojoba seeds [1]. Thus, the jojoba seed lipases seem to have the highest specificity for the most abundant wax esters. The increasing content of glucose during germination suggests that hydrolyzed wax esters serve as substrates for gluconeogenesis, as it was shown by Moreau and Huang [4]. The excess of glucose could be transitorily stored as starch. Our data confirm the previous observations that storage lipid mobilization pattern in jojoba seeds resembles that reported for TAG-accumulating oilseed crops [4, 6]. For seed proteins, the highest rate of mobilization was observed during the first week of germination, which is similar to other plants. High activity of different proteases was observed in the first several days after imbibition in different plant species [27, 28].

In our experiments, the decrease in wax ester amount during germination was accompanied by the reduction of oleosin content in the seed, which occurred mainly after 7 dpg. Such a gradual degradation of oleosins was observed in several plant species, including rapeseed [29], sunflower [30], and sesame [31]. A sunflower thiol-protease is likely to be involved in this process [32]. In *Arabidopsis thaliana,* proteolysis of oleosins precedes lipid degradation, and four out of five oleosins are ubiquitinated before entering the degradation pathway [33]. Further studies are needed to elucidate the mechanism of lipid droplet protein degradation in jojoba. Our results also suggest that at least two isoforms of oleosins with molecular masses of 17 and 19.5 kDa are present in jojoba seeds. It is therefore possible that, as in other angiosperm species, jojoba oleosins belong to both oleosin classes, high (H) and low (L) molecular weight oleosins [34]. The transcriptome analysis of developing jojoba seeds showed that among six jojoba oleosin genes, four were highly expressed in cotyledons during development. The proteins encoded by these genes had high relative abundance in lipid droplet fraction [8], which suggests that they may be involved in the formation of jojoba wax bodies.

The first step of wax esters mobilization is catalyzed by lipases, which hydrolyze a wax ester to a fatty acid and a fatty alcohol. The activity of jojoba lipases was first characterized in wax bodies isolated from jojoba cotyledons [4, 25]. However, in our experiments, the enzymes isolated from the lipid droplet fraction from jojoba seeds at different stages of germination exhibited very low activity. Instead, we observed increasing lipase activity in the microsomal fraction during the first 50 days of germination. This is consistent with Moreau and Huang [4], who reported that 40% of the lipase activity in 20-day-old jojoba seedlings was detected in the wax bodies, 40% in the membrane fraction, and 20% in the soluble fraction. The activity of the lipases in the membrane fraction was attributed to membrane ghosts of the wax bodies, resulting from the remnants of these organelles after wax ester mobilization or their disruption during subcellular fractionation [4]. The increase in oleosin content in our microsomal preparations from jojoba seeds at different stages of germination confirmed the presence of wax bodies’ membranes in this fraction.

In this study, biochemical properties of the jojoba lipases in the microsomal fraction were different than those reported by Huang et al. [25]. The enzymes displayed substantial hydrolase activity in a wide range of temperatures, while in the previous studies the lipases were deactivated by 5 min incubation at 60 °C. On the contrary, our experiments showed that such incubation led to higher enzyme activity, which may be a result of changes in the enzyme microenvironment. We also demonstrated that the jojoba lipases present in the microsomal fractions are not alkaline enzymes as their highest activity was detected at pH 6 and 7 for TAGs and pH ranging from 6.5 to 8 for WEs. These discrepancies might result from different reaction buffers and substrates used in both studies. Huang et al. [25] utilized an artificial substrate, N-methylindoxylmyristate, for biochemical characterization of the jojoba lipase, while in our experiments, 18:1-TAG and 18:1–18:1 WE were used. In addition, here we report high activity of the jojoba lipases towards TAGs, which were hydrolyzed with low efficiency in the experiments performed by Huang et al. [25]. Such activity could be attributed to the possible presence of TAGs in the seeds of jojoba ancestors, however, nowadays, TAGs are localized mostly in the embryonic axis of the jojoba seeds [8, 35]. In our study, we also expanded the spectrum of substrates used by Huang et al. [25]. The jojoba lipases efficiently hydrolyzed almost all tested WEs containing 16:0 or 18:1 and saturated and unsaturated fatty alcohols of different chain lengths.

Compared with plant TAG-lipases characterized so far, the jojoba lipases had similar properties. Most of lipases isolated from seeds of different plant species display the highest activity at pH values 7–8 [36], which was also observed for both TAG- and WE-hydrolyzing activity of the jojoba lipases. The optimal temperature for the jojoba lipases (60 °C) is higher than for the lipases from oilseed plants, such as *Brassica napus* and *Jatropha curcas*, which showed the highest activity at 37 °C [37, 38]. However, the lipases with optimal temperature higher than 50 °C were found in several plant species, including *Amygdalus communis* [39] and *Heliantus annuus* [40]. In addition, Ca^2+^ and Mg^2+^ enhanced the activity of the jojoba enzymes as it was reported for a large number of other lipases [36]. There was a two-fold increase of the lipase activity when these ions were added to the reaction mixture. A similar effect was observed for other oilseed lipases, such as *B. napus* lipase (increase by 64% for Ca^2+^) [38] and *Jatropha curcas* lipase (increase by 130% for Ca^2+^ and by 30% for Mg^2+^) [37].

Despite many studies on lipases, the data on wax ester-hydrolyzing activity of these enzymes is scarce. The ability to hydrolyze wax esters in in vitro assays was reported for lipases from rat, pig and *Pseudomonas fluorescens* [41]. The activity of wax ester hydrolase was also detected in roots of white mustard (*Sinapis alba* L.) [42]. However, the enzyme or enzymes possessing such activity have not yet been identified nor isolated in this species. Lipases from TAG-accumulating plants are usually not tested for wax ester-hydrolyzing activity [36]. It would be valuable to check whether seed lipases of plants used for wax ester production, such as *C. abyssinica* or *C. sativa*, have the ability to hydrolyze wax esters.

In microsomal fraction isolated from germinated jojoba seeds, we also detected wax ester-synthesizing activity, which was inhibited when tetrahydrolipstatin, a lipase inhibitor, was added to the mixture. This indicates that observed de novo synthesis was performed by the jojoba seed lipases in a backward reaction (the enzyme was blocked in both forward and backward reaction by tetrahydrolipstatin). In our enzymatic assays, jojoba lipases preferred a triacylglycerol as a acyl donor over a free fatty acid. The enzymes efficiently synthesized wax esters from free oleic acid (18:1) and different fatty alcohols, which suggests rather broad specificity. It was shown that lipases can catalyze wax ester formation on the surface of substrate emulsions at low concentration of alcohol. According the mechanism proposed by Tsujita et al., incubation of lipases with triacylglycerols or fatty acids leads the formation of an acyl-enzyme intermediate. Since fatty acyl alcohols are more efficient acyl acceptors than water, deacylation of the intermediate results in wax ester synthesis instead of hydrolysis [41]. It is possible that our in vitro assays mimicked these conditions due to the presence of microsomal fractions. However, there is no experimental evidence that lipase-catalyzed wax ester synthesis exists in jojoba.

Wax ester-synthesizing activity was reported for lipases from different organisms, including rat, pig, *Pseudomonas fluorescens* [41], and *Candida* sp. 99–125 [43]. Commercially available lipases, such as Novozym 435 (immobilized lipase B from *Candida antarctica*) and Lipozyme IM (immobilized lipase from *Rhizomucor miehei*), also efficiently catalyze esterification reactions between long chain fatty acids and fatty alcohols [44, 45]. After isolation and further characterization, the jojoba lipases can be useful for industrial synthesis of wax esters due to their high stability and ability to utilize long chain and very long chain alcohols.

Among more than 100 genes encoding lipases, identified in the jojoba genome, 10 had higher expression levels in the developing jojoba seeds compared to the others (Additional file 2: Table S3) [8]. Studies on *Arabidopsis thaliana* showed that the gene encoding SDP1, the major TAG lipase, is expressed predominantly during the seed maturation [46]. Therefore, these 10 genes are promising candidates for the lipases involved in the degradation of storage lipids in jojoba. In addition, since wax esters are mainly localized in the jojoba cotyledons [8], genes with higher expression in this tissue may encode enzymes catalyzing wax ester hydrolysis.

## Conclusions

Our biochemical data suggest that the jojoba lipases may be less unique as previously suspected. The enzymes showed both TAG- and WE-hydrolyzing activity and had an ability to synthesize wax esters. Therefore, the jojoba lipases could share common features with other lipases from oilseed plants. It is necessary to identify, clone and characterize the jojoba lipases to verify this hypothesis. The ability of the jojoba lipases to hydrolyze TAGs with high efficiency may also imply that other seed plant lipases possess wax ester-hydrolyzing activity. Screening for such activity in oilseed crops could be useful in selecting species for wax ester production in seeds.

## Methods

### Reagents

Chemicals used in this study were purchased from Sigma-Aldrich (St. Louis, MO, USA), Merck (Darmstadt, Germany) or Larodan Fine Chemicals (Malmö, Sweden), unless stated otherwise. Non-labeled acyl-CoAs were obtained from Avanti Polar Lipids (Alabaster, AL, USA). Tri-[^14^C]18:1-TAG was purchased from PerkinElmer (Waltham, MA, USA). The [^14^C]acyl-CoAs were synthesized according to the modified method of Sánchez et al. [47] using [^14^C]fatty acids purchased from Biotrend (Cologne, Germany). Radiolabeled wax esters for substrate specificity studies were synthesized using microsomal fractions isolated from *Saccharomyces cerevisiae* transformed with the pVT-URA vector carrying *Marinobacter hydrocarbonoclasticus* wax synthase (MhWS2) gene [48]. In brief, appropriate fatty alcohols (100 nmol/assay) were dissolved in benzene, then added to the freeze-dried yeast microsomal fractions (isolated from the mentioned above yeast transformants). After drying, 0.1 ml of phosphate buffer (pH 6.7) containing 20 nmol of the appropriate [^14^C]acyl-CoA and 0.2 mg bovine serum albumin (BSA) were added. Reaction was carried out for 1 h at 50 °C. Extracted lipids were separated by thin-layer chromatography (TLC) on silica gel 60 plates (Merck, New York, USA) using hexane/diethyl ether/acetic acid (70/30/1, v/v/v) as the solvent system. Silica gel containing wax esters was scraped off and wax esters were extracted according to Bligh and Dyer [49]. The concentration of [^14^C]WEs in the chloroform fraction was determined using Liquid Scintillation Counter (Beckman Coulter, Fullerton, CA, USA).

### Seed material and growth conditions

Seeds of four jojoba accessions (PARL 144, PARL 145, PARL 146 and PARL 147), originated from Arizona, USA were obtained from Janet Caolo-Tanski and Dr. John M. Dyer and from United States Department of Agriculture (USDA). Seeds were collected from plants grown at the site managed by National Arid Land Plant Genetic Resource Unit (NALPGRU; Parlier, CA, USA). Seeds weighing between 600 and 700 mg (accession 145, 146, and 147) or between 900 and 1100 mg (accession 144) were used for studies of wax ester mobilization and isolation of microsomal fractions. The test weight groups were established after determination of average weight of a seed in each accession. This choice allowed to obtain a sufficient amount of seed oil for analysis. The seeds were split into separate beakers and stored in the dry and dark place. Jojoba seeds were germinated in vermiculite in a growth chamber at 28 °C with 16-h photoperiod and collected at 0, 7, 16 and 26 days post germination (dpg) for storage material analysis (Additional file 1: Fig. S1), and at 0, 14, 35 and 50 dpg for microsomal fraction isolation. These time points were selected based on the observation of jojoba germination and on the available literature data [4, 26]. For 0 dpg, mature dry seeds were used. The next stage (7 dpg) was characterized by splitting of the seed coat and emergence of the 1–2 cm radicle. At 16th dpg, the first pair of leaves started to develop on the epicotyl (hypogeal germination) and the radicle reached the length of several cm. The last stage (26 dpg) included plants with several leaves and the developing root system. After 26 dpg, plants still continued growth, however, the seeds remnant were used only for microsomal preparation. For all analyses, the residual seed material without seed cover was used.

### Lipid isolation and analysis

Lipid extraction from individual seeds at each stage of germination (at least 4 seeds from each accession) was carried out using modified Blight and Dyer method [49]. The seeds were chopped into small pieces with a scalpel and homogenized in 11.25 ml of chloroform:methanol (1/2; v/v) using T 25 digital ULTRA-TURRAX (IKA-Werke GmbH & Co. KG, Staufen im Breisgau, Germany). After addition of 3.75 ml of 0.15 M acetic acid, 3.75 ml of chloroform and 3.75 ml of distilled H_2_O, lipids were extracted to chloroform. Extracted lipids were directly analyzed by gas chromatography after methylation and derivatization or first separated on TLC plates in hexane/diethyl ether/acetic acid (70/30/1, v/v/v). Aliquots of total lipid extracts were evaporated to dryness and incubated at 90 °C for 1 h with 2 ml of 0.1 M NaOH in dry methanol. Next, the fatty acid methyl esters and free fatty alcohols were extracted with hexane. The hexane fractions were evaporated to dryness and 0.15 ml of derivatization agent, BSTFA (N,O-bis (trimethylsilyl)trifluoroacetamide), were added to each sample. After 15 min incubation at 70 °C, the fatty acid methyl esters and derivatized fatty alcohols were extracted with hexane and analyzed on Shimadzu GC-2010 equipped with a flame ionization detector (FID) and a 60 m × 0.25 mm CP-WAX 58 CB fused-silica column (Agilent Technologies, Santa Clara, CA, USA). As an internal standard, methyl heptadecanoate was used. The lipids separated by TLC were visualized by brief exposure to iodine vapors. The parts of silica gels containing the analyzed lipid classes were scraped off from the plates, moistened with a small volume of methanol and dried under a stream of nitrogen. The WEs and free alcohols underwent methylation and derivatization as described above. The other lipid classes (not containing long chain alcohols) were methylated at 90 °C for 1 h in 2% (v/v) sulphuric acid in dry methanol, extracted with hexane and analyzed using GC-FID, as described above.

### Protein isolation, SDS-PAGE and immunoblotting

Protein extracts were prepared in a cold room by homogenization of individual seeds at each stage of germination (four seeds from each accession). The seeds were chopped with a scalpel and homogenized three times for 1 min in 20 ml of extraction buffer (0.1 M Tris-HCl, 1% SDS, 1 mM DTT, pH 6.8) using T 25 digital ULTRA-TURRAX (IKA-Werke GmbH & Co. KG). After each homogenization, the samples were centrifuged at 3000 g for 10 min at 4 °C to eliminate the foam. After final centrifugation at 3000 g for 10 min, 1.5 ml aliquots of the supernatant were transferred to new microcentrifuge tubes and kept at − 21 °C until used. Protein concentration was measured using Pierce™ BCA Protein Assay Kit (Thermo Fisher Scientific, Waltham, MA, USA), according to the manufacturer’s instructions. Protein extracts (60–80 μg) were separated by electrophoresis on 4–12% (w/v) NuPAGE gel using NuPAGE MOPS SDS running buffer (Thermo Fisher Scientific). Next, proteins were transferred onto nitrocellulose membrane in NuPAGE transfer buffer (Thermo Fisher Scientific) and analyzed using BM chemiluminescence Western blotting kit (Mouse/Rabbit) (Roche Diagnostics GmbH, Mannheim, Germany) and ChemiDoc XRS+ System (BioRad, Hercules, CA, USA), according to the manufacturer’s instructions. The primary rabbit antibody recognizing oat oleosins [50] was used at a dilution of 1:10,000.

### Carbohydrate isolation and analysis

Four individual seeds of accession 147 from different stages of germination were macerated in liquid nitrogen, dried at 80 °C for 24 h, and ground up to fine powder with a mortar and a pestle. The content of glucose and starch was determined in 50 mg of powder of each seed. The powder was first extracted three times with 5 ml of 80% ethanol by incubation at 85 °C for 10 min. After each extraction, the samples were centrifuged at 3000 g for 10 min and combined supernatants were used for glucose analysis using Megazyme Glucose Determination Reagent (glucose oxidase/peroxidase; GOPOD) as described in the manual for D-Glucose Assay Kit (GOPOD Format) (Megazyme, Bray, County Wicklow, Ireland). The content of starch was measured in the residual pellet according to the manufacturer’s instructions using Total Starch Kit (Megazyme), based on the method of McCleary et al. [51].

### Microsomal membrane preparation and enzyme assays

Microsomal fractions for preliminary assessment of lipase activity were prepared from four individual seeds from each fraction. For biochemical characterization of lipases, microsomal fractions were prepared from germinated jojoba seeds (35 dpg) of four accessions mixed together (two seeds from each accession). All steps were carried out in a cold room. Seeds or cotyledons were chopped into small pieces with a scalpel and ground in a glass homogenizer in ice-cold 0.1 M potassium phosphate buffer, pH 7.2, containing 0.33 M sucrose, 1000 U/ml catalase and 1 mg/ml BSA. The next steps were performed as described in Stymne and Stobart [52].

Lipases activity was measured in assays with tri-[^14^C]18:1-TAG or radiolabeled WEs (20 nmol of [^14^C]TAG or [^14^C]WE was used if not specified differently under the tables or figures). TAG or WEs were dissolved in 19 μl benzene and added to the freeze-dried microsomes (corresponding to 11 μg of microsomal protein). After immediate evaporation of the solvent, the buffer (0.1 M phosphate buffer or 0.1 M HEPES) was added. The assays (final volume 100 μl) were incubated for appropriate time with shaking (1250 rpm) at a given temperature. At the end of incubation, lipids were extracted from reaction mixtures into chloroform according to Bligh and Dyer [49] and separated on TLC plates in hexane/diethyl ether/acetic acid (70/30/1; v/v/v). Separated products of the lipases activity were visualized and quantified directly on the TLC plate using electronic autoradiography (Instant Imager, Packard Instruments).

Assays for measuring wax ester-synthesizing activity of jojoba lipases were performed as described above using different acyl donors and fatty alcohols as substrates. 20 μmol of tetrahydrolipstatin (Orlistat) and 0.7 nmol of DTNB (5,5-dithio-bis-(2-nitrobenzoic acid)) were added to the reaction mixture to assess their influence on the lipase activity.

### Statistical analysis

For statistical analysis, the Statistica 13 software from Statsoft was used. The contents of fatty acids and fatty alcohols in the seeds of jojoba accessions during germination were compared using an one-way analysis of variance, followed by post-hoc Tukey’s test. Student’s t-test was used to compare efficacy of wax ester synthesis by microsomal fractions of germinated jojoba seeds from different substrates. For both analyses, *p* values less than 0.01 or 0.05 were considered significant.

## Supplementary Information

**Additional file 1: Fig. S1.** Different stages of jojoba germination and post-germinative growth. **Fig. S2.** Changes in starch and glucose content in germinating jojoba seeds. **Fig. S3.** Immunoblot analysis of oleosin content in the microsomal fractions isolated from jojoba seeds (accession 147) at different stages of germination. **Fig. S4.** Time course of TAG hydrolysis in phosphate buffer and HEPES buffer. **Fig. S5.** The effect of Ca^2+^ and Mg^2+^ on the jojoba seed lipase activity towards 18:1-TAG. **Fig. S6.** The original uncropped version of Fig. 2.

**Additional file 2: Table S1.** The relative content of main fatty acids (FA) and fatty alcohols (FA-OH) in lipids of mature jojoba seeds (% of total FA and FA-OH). **Table S2.** Lipase activity in the microsomal fractions isolated from jojoba seeds at different stages of germination. **Table S3.** List of 10 genes encoding putative jojoba lipases with the highest gene expression levels during seed development.

## Data Availability

The datasets used and/or analysed during the current study are available from the corresponding author on reasonable request.

## Abbreviations

FA: fatty acid
FA-OH: fatty alcohol
dpg: days post germination
TAG: triacylglycerols
WE: wax esters
