# Supplementary material for: Lipases of germinating jojoba seeds efficiently hydrolyze triacylglycerols and wax esters and display wax ester-synthesizing activity
Source: BMC Plant Biol. 2021 Jan 19;21:50. doi: 10.1186/s12870-020-02823-4 (PMC7814598; doi:10.1186/s12870-020-02823-4)
Supplement: Supplementary file 1 — Additional file 1: Fig. S1. Different stages of jojoba germination and post-germinative growth. Fig. S2. Changes in starch and glucose content in germinating jojoba seeds. Fig. S3. Immunoblot analysis of oleosin content in the microsomal fractions isolated from jojoba seeds (accession 147) at different stages of germination. Fig. S4. Time course of TAG hydrolysis in phosphate buffer and HEPES buffer. Fig. S5. The effect of Ca2+ and Mg2+ on the jojoba seed lipase activity towards 18:1-TAG. Fig. S6. The original uncropped version of Fig. 2. [file 12870_2020_2823_MOESM1_ESM.pdf]

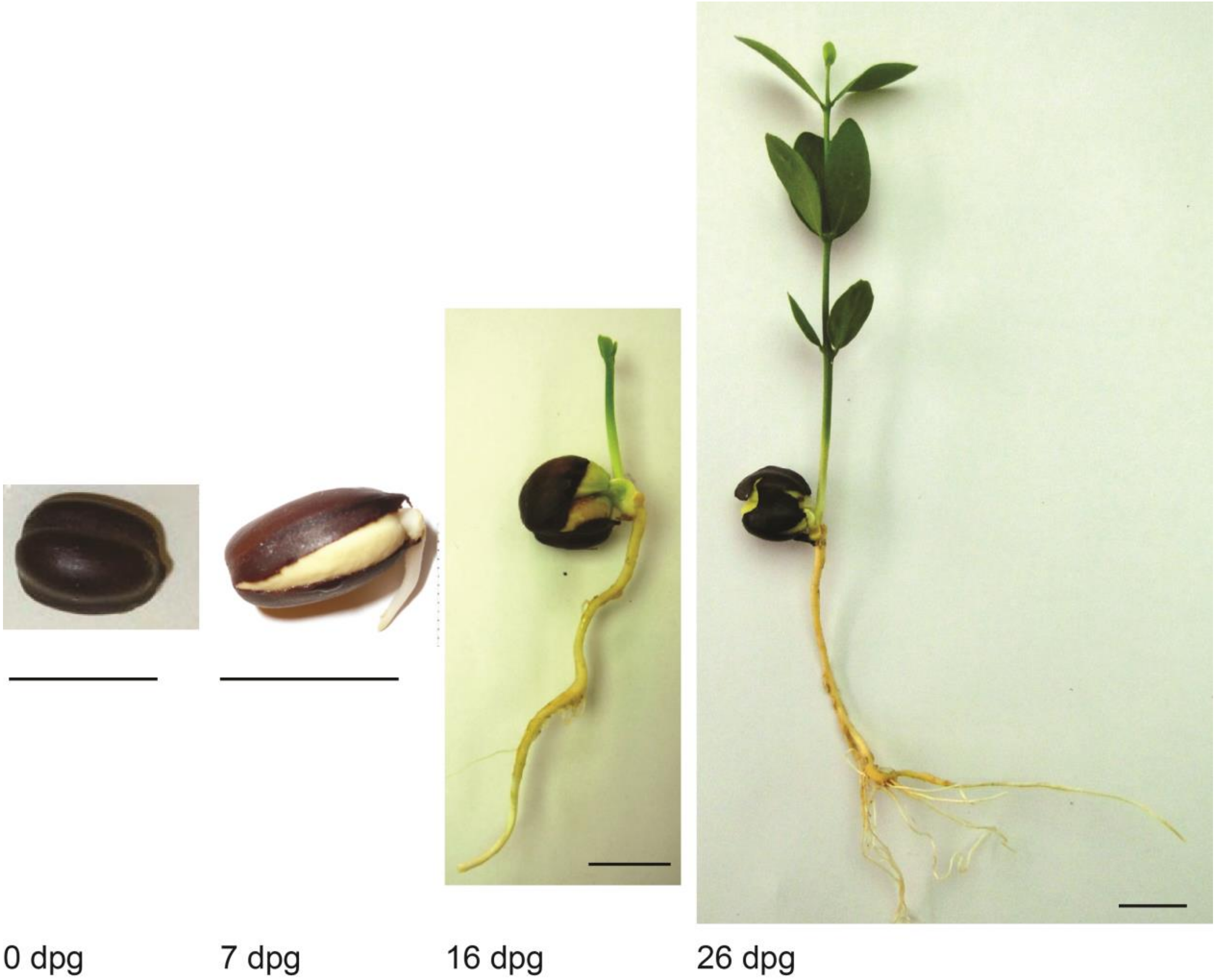

Figure S1. Different stages of jojoba germination and post-germinative growth. Scale bar, 1.0 cm.

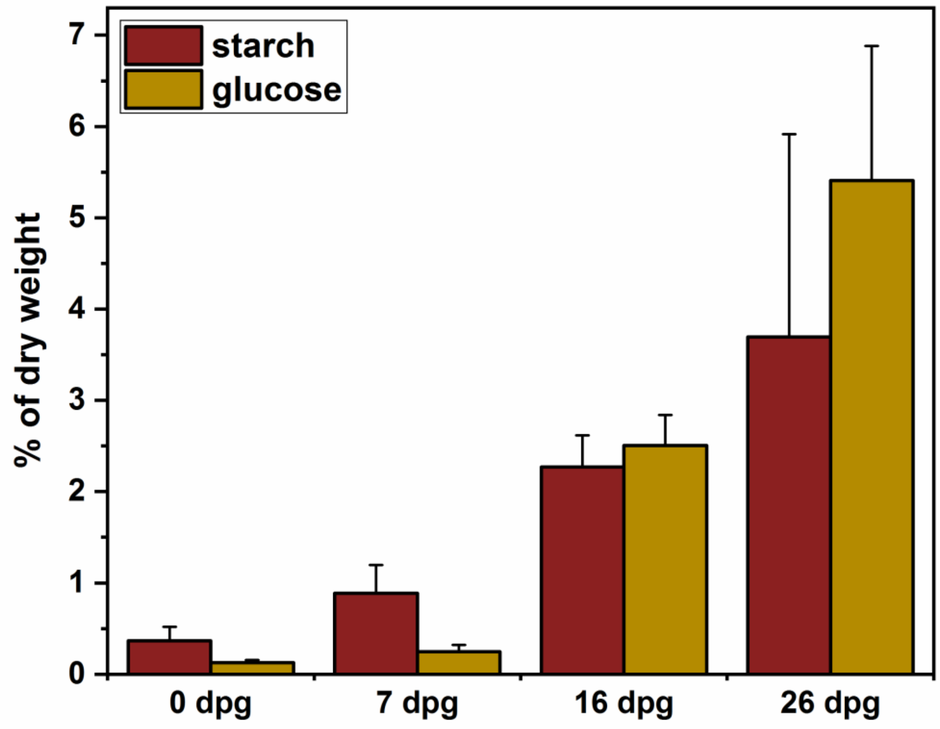

Figure S2. Changes in starch and glucose content in germinating jojoba seeds. Data represent the mean of four biological replicates (accession 147) and error bars show standard deviation.

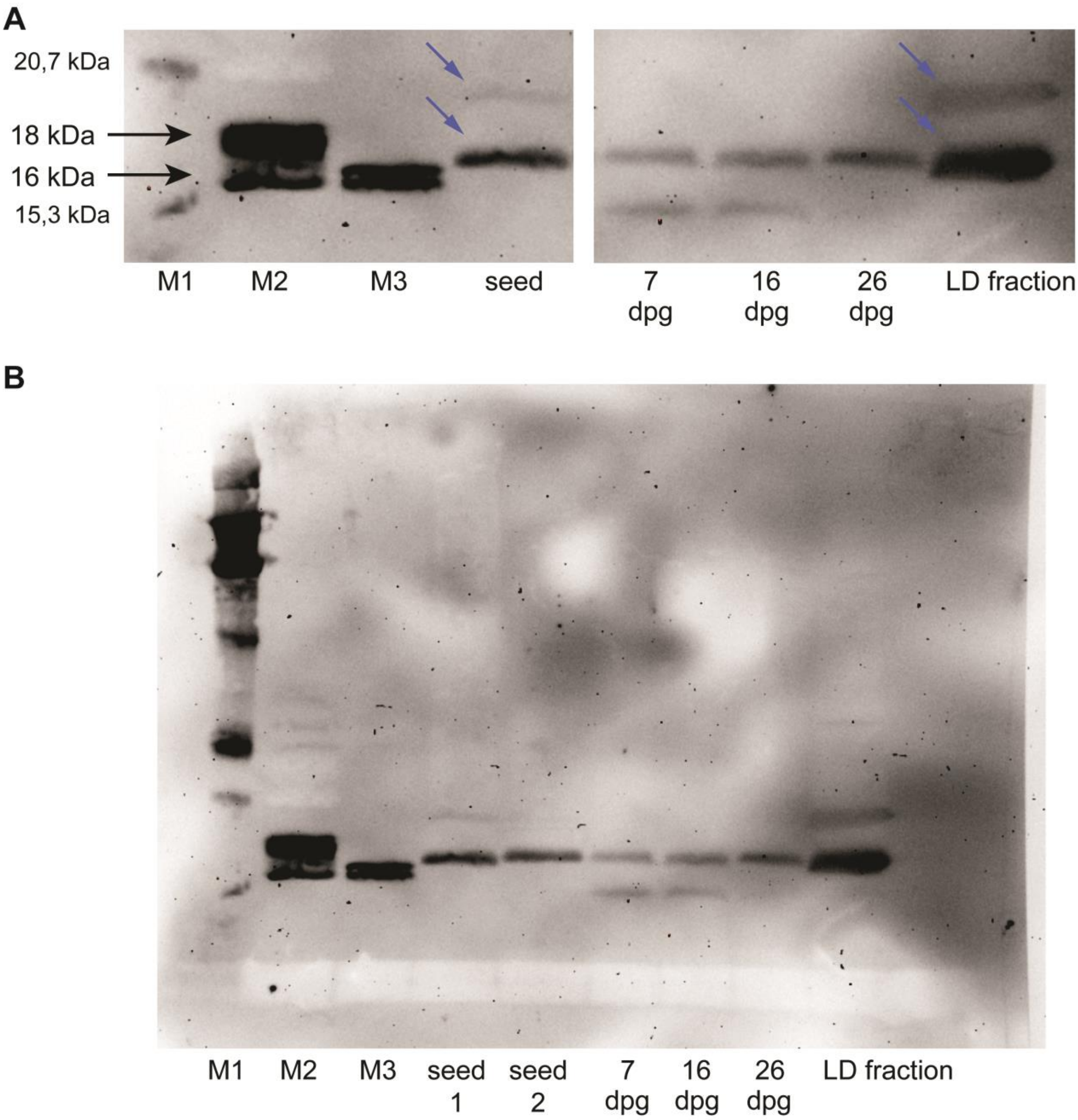

Figure S3. Immunoblot analysis of oleosin content in the microsomal fractions isolated from jojoba seeds (accession 147) at different stages of germination. A. M1: protein marker, M2: oleosins from maize, M3: oleosins from oat, seed: total protein extract from mature jojoba seeds, LD fraction: lipid droplet (wax body) fraction isolated from mature jojoba seeds. Numbers to the left indicate the positions of molecular mass markers. The black arrows indicate two isoforms of the maize oleosins. The blue arrows indicate two isoform of the jojoba oleosins (app. 17 kDa and 19.5 kDa). B. The original uncropped version.

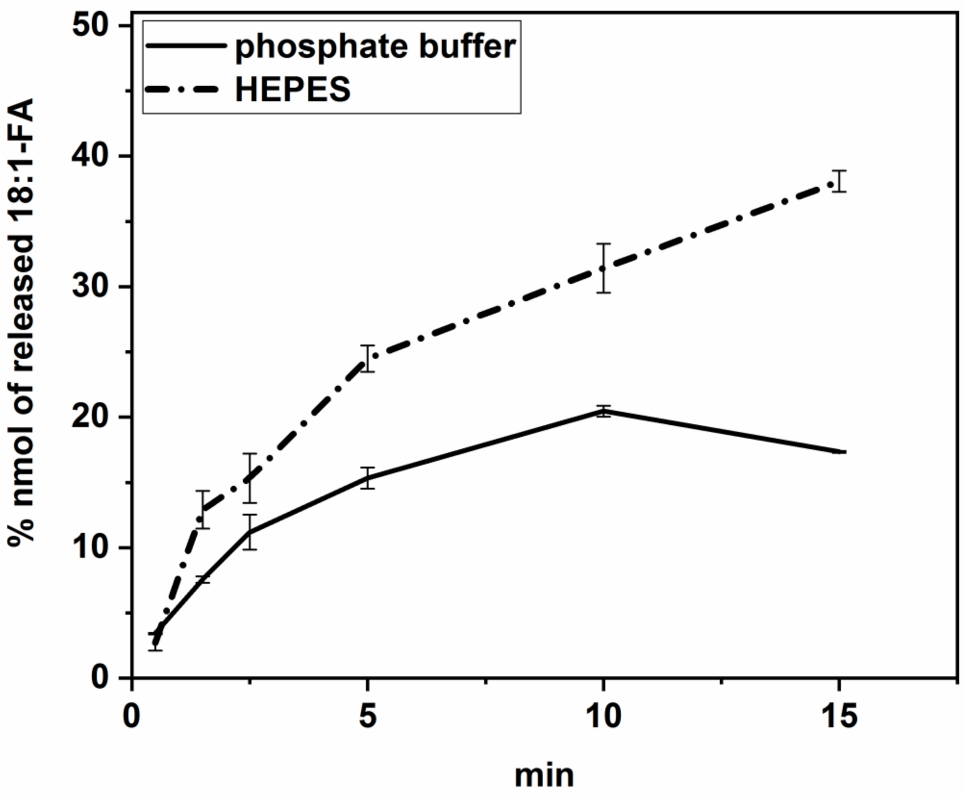

Figure S4. **Time course of TAG hydrolysis in phosphate buffer and HEPES buffer.** Data represent mean values and error bars show the range of duplicates. Assay condition: aliquots (2.5 nmol of endogenous PC) of microsomal fraction mixture isolated from two individual jojoba seeds from each accession (35 dpg); 20 nmol of [^{14}C]18:1-TAG added to freeze-dried microsomes in 19 µl benzene; benzene evaporation, and addition of 100 µl 0.1 M phosphate buffer (pH 7.0) or 0.1 M HEPES buffer (pH 7.); incubation at 60°C.

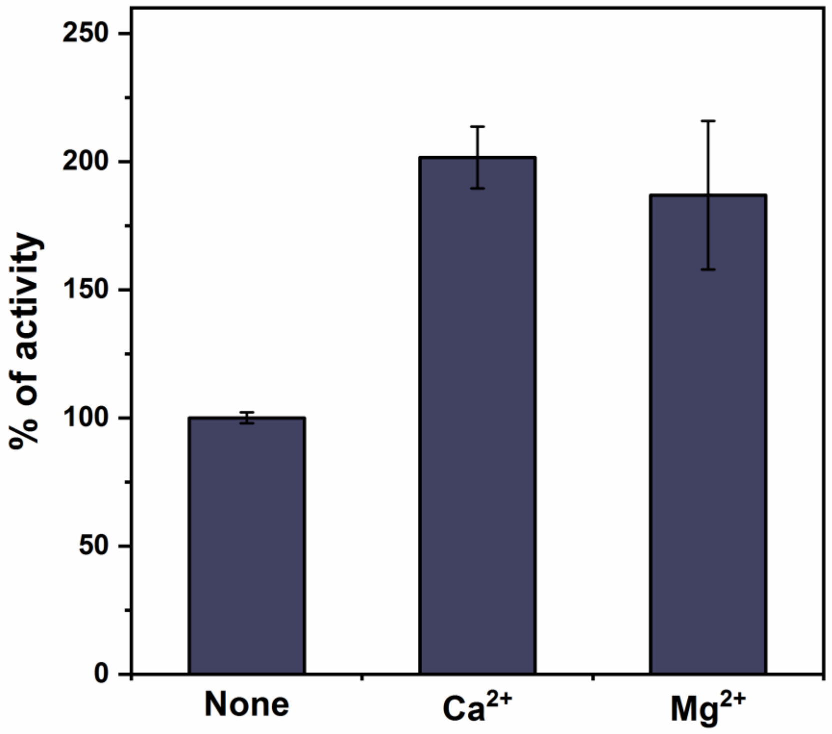

Figure S5. The effect of Ca^{2+} and Mg^{2+} on the jojoba seed lipase activity towards 18:1-TAG.

Data represent mean values and error bars show the range of duplicates. Assay condition: aliquots (2.5 nmol of endogenous PC) of microsomal fraction mixture isolated from two individual jojoba seeds from each accession (35 dpg); 20 nmol of [^{14}C]18:1-TAG added to freeze-dried microsomes in 19 µl benzene; benzene evaporation, and addition of 100 µl 0.1 M HEPES buffer (pH 7.0) or 0.1 M HEPES buffer containing 5 mM $CaCl_2$ or 5 mM $MgCl_2$; 5 min incubation at 60°C.

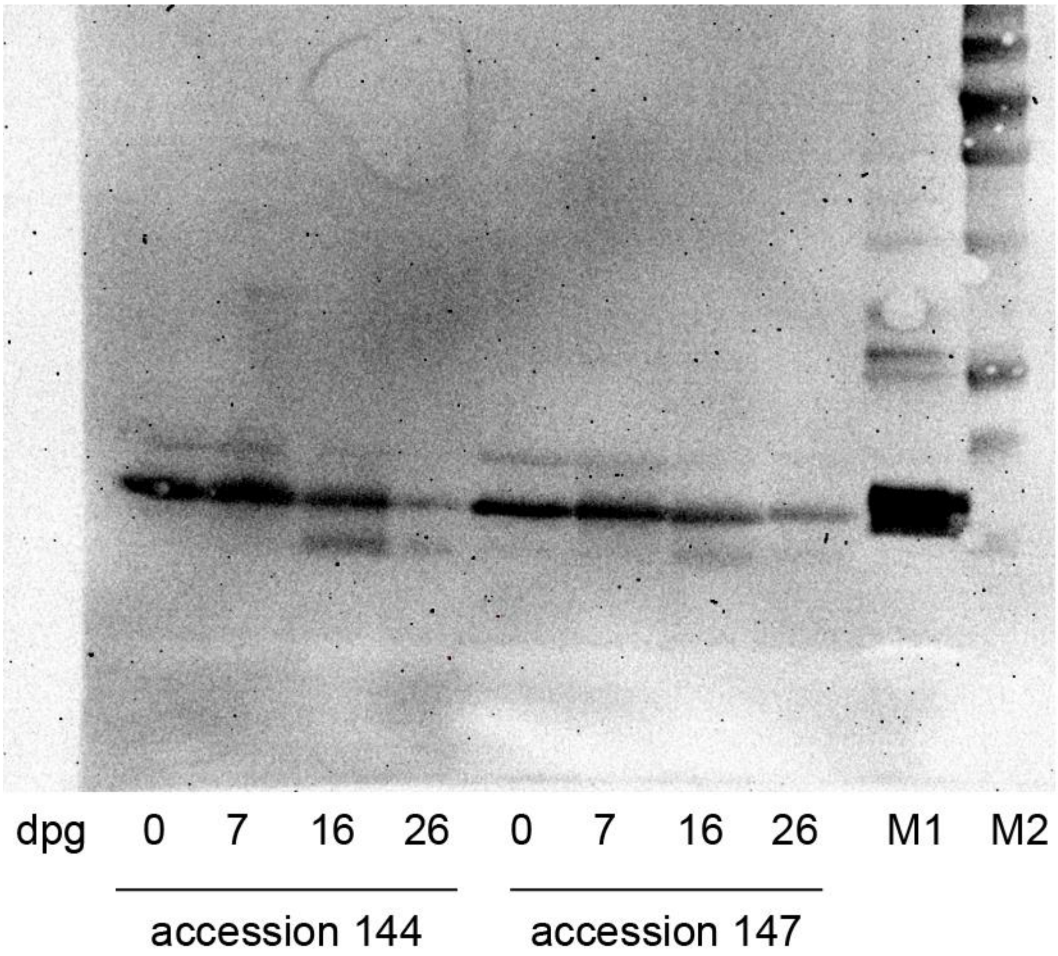

Figure S6. The original uncropped version of Figure 2. Immunoblot analysis of oleosin content in total protein extracts of jojoba seeds during germination. M1: oleosins from maize, M2: protein marker.
