# Supplementary material for: Lipases of germinating jojoba seeds efficiently hydrolyze triacylglycerols and wax esters and display wax ester-synthesizing activity
Source: BMC Plant Biol. 2021 Jan 19;21:50. doi: 10.1186/s12870-020-02823-4 (PMC7814598; doi:10.1186/s12870-020-02823-4)
Supplement: Supplementary file 2 — Additional file 2: Table S1. The relative content of main fatty acids (FA) and fatty alcohols (FA-OH) in lipids of mature jojoba seeds (% of total FA and FA-OH). Table S2. Lipase activity in the microsomal fractions isolated from jojoba seeds at different stages of germination. Table S3. List of 10 genes encoding putative jojoba lipases with the highest gene expression levels during seed development. [file 12870_2020_2823_MOESM2_ESM.pdf]

Table S1. The relative content of main fatty acids (FA) and fatty alcohols (FA-OH) in lipids of mature jojoba seeds (% of total FA and FA-OH).

FA/FA-OH	Accession 144	Accession 145	Accession 146	Accession 147
16:0	0.93 ± 0.08	0.68 ± 0.12	0.88 ± 0.12	0.85 ± 0.20
18:1	6.50 ± 0.57	5.89 ± 0.65	7.14 ± 0.86	8.29 ± 2.38
20:1	35.90 ± 0.39	37.25 ± 0.23	35.83 ± 0.74	35.49 ± 1.31
22:1	6.02 ± 0.29	5.60 ± 0.72	5.71 ± 0.51	4.93 ± 1.14
24:1	0.65 ± 0.15	0.58 ± 0.20	0.45 ± 0.16	0.45 ± 0.20
18:1-OH	0.72 ± 0.07	0.62 ± 0.14	0.55 ± 0.11	0.79 ± 0.35
20:1-OH	23.67 ± 1.37	25.41 ± 2.82	25.08 ± 2.47	27.27 ± 4.46
22:1-OH	22.01 ± 1.06	20.74 ± 1.92	21.24 ± 1.84	19.34 ± 3.78
24:1-OH	3.61 ± 0.43	3.23 ± 1.04	3.13 ± 0.75	2.60 ± 1.03

Data represent the mean of four biological replicates ± standard deviation.

Table S2. Lipase activity in the microsomal fractions isolated from jojoba seeds at different stages of germination.

Substrate added (1 nmol/assay)	% of added radioactivity in free fatty acids fraction			
	Germination time			
	0 day	14 days	35 days	50 days
Triacylglycerol: Tri-[^{14}C]18:1-TAG	traces	6.2 ± 0.7	44.8 ± 4.4	67.3 ± 4.2
Wax ester: 20:1-OH-[^{14}C]18:1-FA	traces	2.2 ± 0.7	11.7 ± 2.5	18.5 ± 1.0

Data represent the mean of four to six biological replicates ± standard deviation. Assay condition: aliquots (2.5 nmol of endogenous PC) of microsomal fractions of jojoba germinating seed; substrates added to freeze-dried microsomes in 19 µl benzene; benzene evaporation and addition of 100 µl 0.1 M phosphate buffer (pH 7.2); 15 min incubation at 35°C.

Table S3. List of 10 genes encoding putative jojoba lipases with the highest gene expression levels during seed development.

Gene ID	SwissProt/Pfam description	Tissue with the highest expression level
Sc01g0002810	Lipase (class 3)	Cotyledons
Sc04g0004940	GDSL esterase/lipase	Cotyledons
Sc04g0009590	GDSL esterase/lipase	Cotyledons
Sc07g0004640	GDSL esterase/lipase	Cotyledons
Sc10g0010100	GDSL esterase/lipase	Cotyledons
Sc12g0010090	GDSL esterase/lipase	Seed coat
Sc12g0010880	GDSL esterase/lipase	Cotyledons
Sc16g0010020	GDSL esterase/lipase	Seed coat
Sc17g0002600	GDSL esterase/lipase	Seed coat

The genes were selected based on the data presented by Sturtevant et al. (2020) in Supplementary materials (Table S16 and S17) [8].
